# Multi-Drug Resistance in Bacterial Genomes—A Comprehensive Bioinformatic Analysis

**DOI:** 10.3390/ijms241411438

**Published:** 2023-07-14

**Authors:** Célia P. F. Domingues, João S. Rebelo, Francisco Dionisio, Teresa Nogueira

**Affiliations:** 1 cE3c—Centre for Ecology, Evolution and Environmental Changes & CHANGE, Global Change and Sustainability Institute, Faculdade de Ciências, Universidade de Lisboa, 1749-016 Lisboa, Portugal; celiapfd@hotmail.com (C.P.F.D.); joaorebelo_4@hotmail.com (J.S.R.); 2 INIAV—National Institute for Agrarian and Veterinary Research, 2780-157 Oeiras, Portugal

**Keywords:** antibiotic resistance, multi-drug resistance, plasmid, co-selection, genomics, evolution

## Abstract

Antimicrobial resistance is presently one of the greatest threats to public health. The excessive and indiscriminate use of antibiotics imposes a continuous selective pressure that triggers the emergence of multi-drug resistance. We performed a large-scale analysis of closed bacterial genomes to identify multi-drug resistance considering the ResFinder antimicrobial classes. We found that more than 95% of the genomes harbor genes associated with resistance to disinfectants, glycopeptides, macrolides, and tetracyclines. On average, each genome encodes resistance to more than nine different classes of antimicrobial drugs. We found higher-than-expected co-occurrences of resistance genes in both plasmids and chromosomes for several classes of antibiotic resistance, including classes categorized as critical according to the World Health Organization (WHO). As a result of antibiotic-resistant priority pathogens, higher-than-expected co-occurrences appear in plasmids, increasing the potential for resistance dissemination. For the first time, co-occurrences of antibiotic resistance have been investigated for priority pathogens as defined by the WHO. For critically important pathogens, co-occurrences appear in plasmids, not in chromosomes, suggesting that the resistances may be epidemic and probably recent. These results hint at the need for new approaches to treating infections caused by critically important bacteria.

## 1. Introduction

Antibiotics are one of the most important discoveries in public health and medical science as they have revolutionized the treatment of infectious diseases and limited the spread of pathogens. The development of new drugs and their use have saved millions of lives and considerably increased average life expectancy and human and animal welfare [1]. Nevertheless, the inappropriate and intensive use of antibiotics since their discovery have led to a worrying crisis of antibiotic resistance which is currently considered as one of the top 10 global public health threats by the World Health Organization (WHO) [2].

In the practice of human and veterinary medicine, antibiotics are abundantly administered and delivered into the environment, selecting resistant bacteria in both habitats. This contamination drives the abundant detection of resistant bacteria in a wide range of environments, such as air [3], clinical wastewater [4], water [5,6,7,8], soil [9], animal feces [10], and sewage waste [11], making these environments critical reservoirs of antibiotic resistance genes [12]. Also contributing to the spread of antibiotic resistance genes are bacteriophages that acquire the genes during transduction [13]. An example is coliphages that transduces genes encoding mainly for kanamycin and chloramphenicol resistances (but also for tetracycline) to *Escherichia coli* [14].

Meanwhile, the co-selection of resistance genes with genes coding for other characteristics has occurred, for example, such is the case for heavy metal tolerance genes [15] and virulence genes [16]. Co-selection of antibiotic resistance with heavy metals has already been observed in metagenomic samples as in animals [17,18], soils [19], water [20], and also at the genomic level, for instance, in bacteria of the genus *Aeromonas* [21] and *Vibrio parahaemolyticus* [18].

Regarding co-selection with virulence genes in metagenomics samples, in recent years, Escudeiro et al. made the intriguing observation that there is a positive correlation between resistance and virulence genes’ diversities in microbiomes [16]. This positive correlation occurs in both types of microbiomes studied: environmental and human intestinal [16]. With computer simulations, we have recently shown that social contact between people drives the positive correlation between virulence genes’ diversity and resistance genes’ diversity in human microbiomes [22,23]. In time, both genes’ types accumulate in metagenomes. We arrived at the a priori counter-intuitive conclusion that the microbiomes with a higher diversity in both genes pertain to people that took antibiotics a longer time ago. People who recently took antibiotics have resistance and virulence genes at low levels of diversity in their metagenomes [22,23].

At the genomic level, for reference genomes, it was shown that there are co-occurrences of some resistance classes and some virulence classes, for example, between fusidic acid resistance and type VII secretion systems [24]. There is also evidence of this association in several pathogenic species [25,26]. For example, *Enterococcus faecalis* can transfer resistance and virulence genes to other bacteria through horizontal gene transfer [27].

In addition to the danger of co-selection of resistances with other factors such as those mentioned above, bacteria with resistance to several antibiotic categories have already been observed [28]. This presence of multiple resistance genes in the same bacterium can result from a co-selection process. This selection of multiple resistances has already been demonstrated in pig microbiomes [29] and, more recently, in children’s digestive tract [30]. Recently, Martiny et al. analyzed 214,095 metagenomic datasets with samples from different environments [31]. The authors showed several co-occurrences between resistance gene classes, some of which involve critically important antibiotics for humans as defined by the WHO [2]. According to the WHO, an antibiotic is critically important if it meets the following two distinct criteria: (i) it must be a drug of last resort for the treatment of bacterial infections in humans; and (ii) the infections must potentially be of non-human origin or caused by bacteria with resistance genes obtained from non-human hosts.

Antibiotic resistance is a serious problem that directly affects human health. Due to the high level of resistance observed and the evidence of co-occurrence between resistance classes in several metagenomic samples, the objectives of this study are: (1) to analyze which resistances are present in the reference bacterial genomes obtained from the RefSeq database, and (2) to verify if there are co-occurrences between different resistance classes. We used a dataset previously prepared by our group composed of more than 16,000 closed bacterial genomes [24]. We studied co-occurrence on the plasmid, chromosome, or both locations, using the classification by classes of antibiotics defined by the ResFinder database. ResFinder includes the category «disinfectants» as an antibiotic resistance class.

Plasmids encoding resistance to more than one class of antibiotics have already been identified [32,33,34]. These resistances could transfer between bacteria of the same or different species through horizontal gene transfer [35,36].

In this study, we aim to understand the existence of co-occurrences of antibiotic resistance genes in the same cell as well as the possibility of joint mobilization of resistance to more than one class of antibiotics, considering that plasmids can move to other cells.

## 2. Results

To analyze the co-occurrences between classes of resistance genes, we used the dataset previously prepared by our group [24]. This dataset consists of 16,622 closed bacterial genomes, whose replicons are classified as belonging to either the chromosome or the plasmid. Each replicon can belong to only one of these categories. We note that we cannot differentiate how many plasmids each genome has nor distinguish whether genes from the same bacterium are located on the same or different plasmids.

### 2.1. Specific versus Nonspecific Proteins

**Figure 1:**
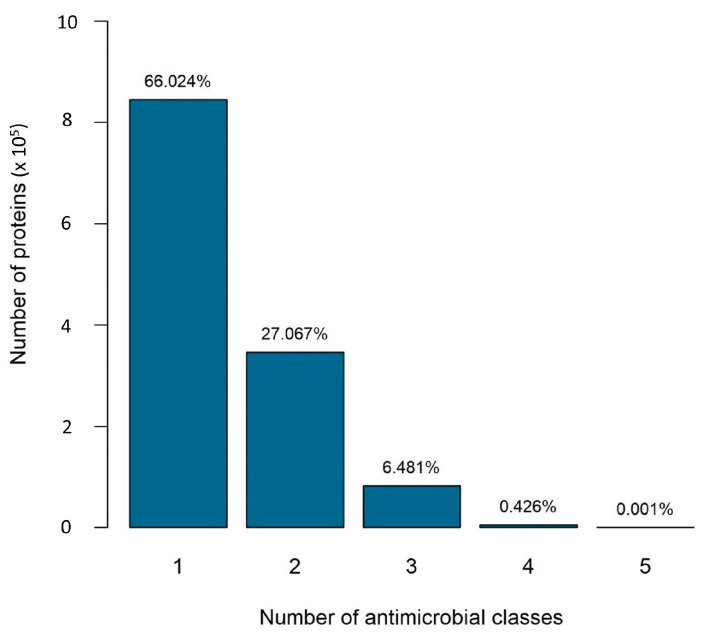
Proteins associated with different numbers of antimicrobial classes. Bars represent the number of proteins associated with x resistance classes. The *x*-axis indicates the number of resistance classes, and the *y*-axis indicates the number of proteins.

Analyzing the BLASTP alignment results against the ResFinder database, we observed that sometimes the same protein query was hit with more than one antibiotic resistance class. Likely, these proteins are associated with less specific antibiotic resistance mechanisms, such as efflux pumps. These types of mechanisms may increase the co-occurrences of resistance classes. In these cases, bacteria are resistant to two or more antibiotics, not because they have a specific resistance to each one, but because they code for a mechanism that confers resistance to multiple antibiotics. To understand the extent of this effect, we counted how many proteins are associated with only one resistance class and how many are associated with more than one. We realized that 66% of the proteins are associated with only one resistance class, and no proteins are associated with more than five classes (Figure 1).

From here on, we consider two scenarios:

Scenario 1 considers specific proteins: those that confer resistance to a single antibiotic resistance class;
Scenario 2 considers all proteins: those that confer resistance to one or more classes.

Therefore, scenario 1 (specific proteins) takes into account 844,982 proteins, and scenario 2 (all proteins) considers all the 1,279,810 proteins (therefore, scenario 2 includes the proteins of scenario 1).

### 2.2. The Number of Antibiotic Resistance Classes in Bacterial Genomes

To understand which resistance classes are the most and the least present in bacterial genomes, we counted the number of genomes encoding proteins conferring resistance for each antibiotic resistance class. We considered the two scenarios mentioned above: genes encoding proteins conferring resistance to a single antibiotic class versus one to five classes (Table 1).

In the first scenario (specific proteins), we did not identify resistance to aminoglycosides and rifampicin in any genomes. When considering scenario 2 (all proteins), we have identified resistance to these two antibiotics in 887 genomes, which may be associated with efflux pumps (a nonspecific mode of resistance common to these two antibiotics).

In both scenarios, resistance to disinfectants, glycopeptides, macrolides, and tetracyclines are present in more than 95% of the genomes. On the other hand, we observe a strong presence of genes conferring ressitance to oxazolidinones and quinolones considering the second scenario (all proteins), as opposed to when considering only the resistance genes conferring resistance to a single antibiotic class.

**Figure 2:**
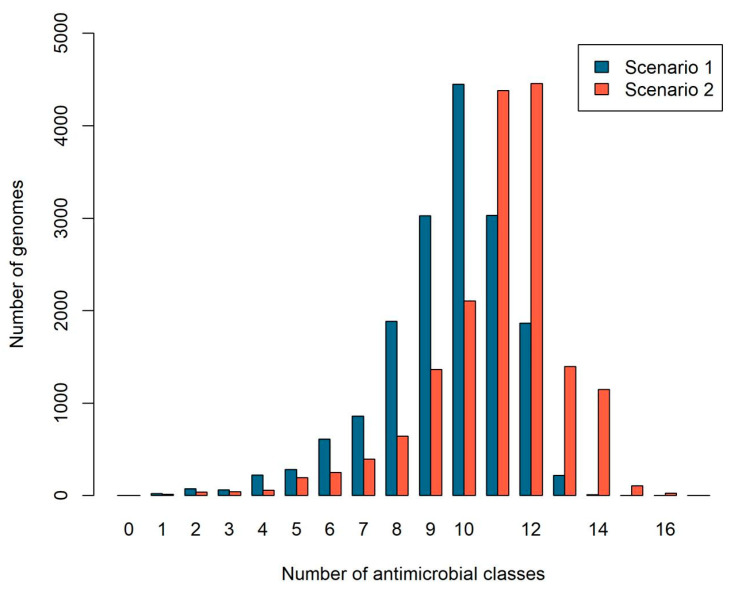
Number of antimicrobial classes per genome. Blue bars represent counts considering scenario 1 (specific proteins) and red bars represent counts considering scenario 2 (all proteins). The *x*-axis indicates the number of antimicrobial classes, and the *y*-axis indicates the number of genomes.

We then counted the number of different resistance classes per genome, considering scenarios 1 and 2 (Figure 2). On average, each genome has (mean ± sd) 9.50 ± 1.92 resistance classes considering scenario 1 (specific proteins) and 10.95 ± 1.99 considering scenario 2 (all proteins).

### 2.3. Co-Occurrence of Antibiotic Resistance Classes

**Figure 3:**
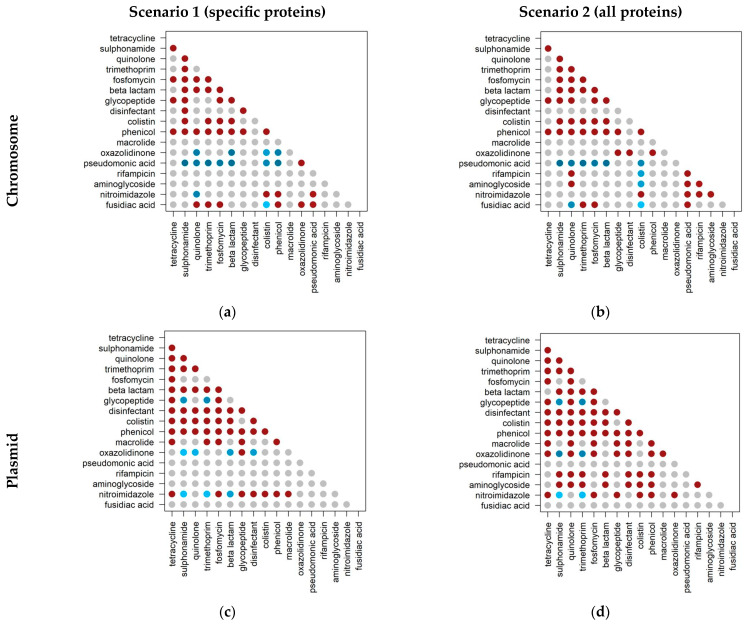
Co-occurrence of antimicrobial classes in chromosomes and plasmids. The panels correspond to: (**a**) scenario 1 (including proteins conferring resistance to one class of antibiotics) in chromosome; (**b**) scenario 2 (including proteins conferring resistance to all classes of antibiotics) in chromosome; (**c**) scenario 1 in plasmid; and (**d**) scenario 2 in plasmid. Red and blue circles represent cases where the observed co-occurrence is higher and lower than expected, respectively (binomial test, *p* < 0.001). Darker circles represent observed/expected values further away from zero. Gray circles represent cases where co-occurrence is not significantly different from the expected value.

We then assessed the co-occurrence of antibiotic resistance genes in the same genome. To perform this analysis, we constructed a contingency table where each value corresponds to the number of genomes containing each combination of resistance classes. We performed a binomial test to test whether the co-occurrence among classes differs from the expected value. The heatmaps represent the observed/expected ratio, where values above 1 represent cases where the observed value is higher (red circles) or lower (blue circles) than expected. The circles are grey if the observed and the expected values are not statistically different (Figure 3). We performed this analysis considering the specific proteins versus all proteins. Since there are no counts for proteins associated with resistance to rifampicin or aminoglycosides, the corresponding points are in grey. We split this analysis by genomic location.

Figure 3 shows 50 co-occurrences above the expected value in plasmids and 39 in chromosomes, considering the first scenario (specific proteins). Considering the second scenario (all proteins), there are 72 co-occurrences above the expected value in plasmids and 44 in chromosomes.

According to the World Health Organization (WHO), macrolide, quinolone, glycopeptide, colistin (polymyxins), and β-lactam (cephalosporins) are categorized as antibiotics of critical importance to human health. Within these categories, and considering the first scenario (specific proteins), the combinations quinolone—β-lactams and colistin—β-lactams have co-occurrences above the expected value on both chromosome and plasmid.

### 2.4. Co-Occurrence of Antibiotic Resistance Classes in Antibiotic-Resistant Priority Pathogens

**Figure 4:**
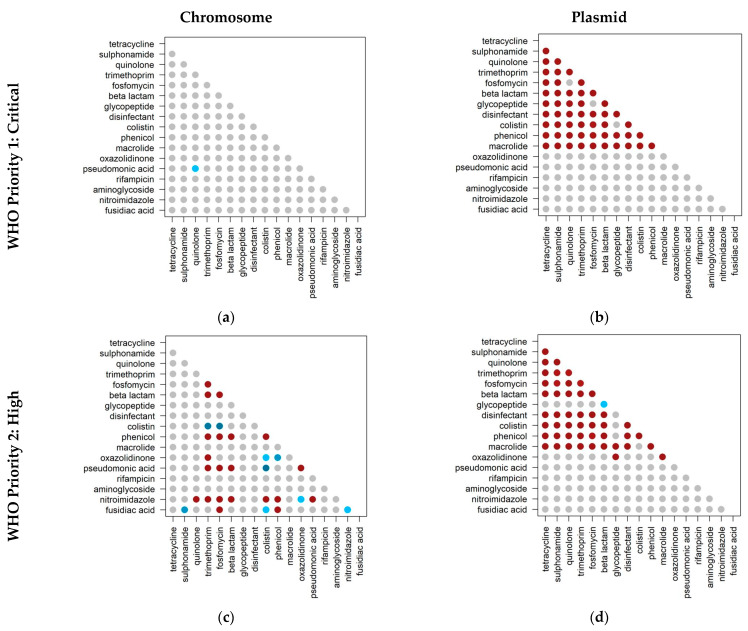

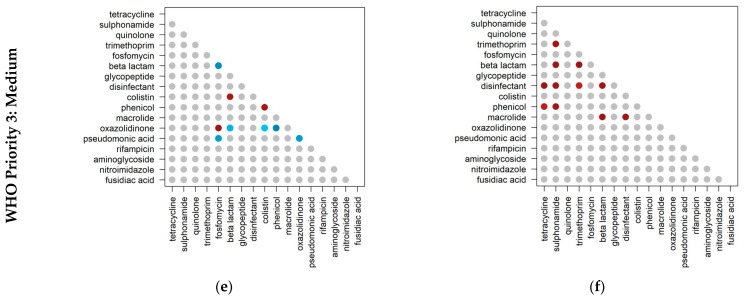
Co-occurrences of antibiotic resistance classes in antibiotic-resistant priority pathogens, considering specific proteins (including proteins conferring resistance to one class of antibiotics). Priorities refer to the categorization of priorities according to the WHO (critical, high, and medium). The panels correspond to: (**a**) priority 1 on chromosomes; (**b**) priority 1 on plasmids; (**c**) priority 2 on chromosomes; (**d**) priority 2 on plasmids; (**e**) priority 3 on chromosomes; and (**f**) priority 3 on plasmids. Red and blue circles represent cases where the observed co-occurrence is higher and lower than expected, respectively (binomial test, *p* < 0.001). Darker circles represent observed/expected values farther from zero. Gray circles represent cases where co-occurrence is not significantly different from the expected value.

In addition to categorizing antibiotics of critical importance to human health, the World Health Organization also identifies antibiotic-resistant priority pathogens. These pathogens are divided by priority, namely: (1) Critical; (2) High; and (3) Medium. To understand the distribution of co-occurrences considering these different priorities, we used the feature tables to select only the genomes belonging to species in each of these categories, considering scenario 1 (specific proteins). We were left with 2366 genomes for priority category 1, 2249 genomes for priority category 2, and 716 genomes for priority category 3. Our results show that co-occurrences for priority category 1 appear higher than expected, considering only the genomic location ‘plasmid’ (Figure 4).

## 3. Discussion

To our knowledge, this is the first large-scale study aiming to identify the co-occurrence of antibiotic resistance gene classes and multi-drug resistance in complete, closed bacterial genomes. We used a dataset prepared by our group, consisting of the orthologues of antibiotic resistance genes from more than sixteen thousand genomes obtained from the RefSeq database, with replicons classified as belonging to the chromosome or plasmid [24].

Characterizing our dataset, we found several proteins were hit with more than one class of antibiotic resistance genes. This indicates an association of these proteins with nonspecific resistance mechanisms. In our dataset, 66% of the proteins confer resistance for only one antibiotic resistance class, and the remaining percentage confer for two (27%), three (6.5%), four (0.43%), or at most five (0.001%) different resistance classes. For this reason, we considered two distinct scenarios: scenario 1 considers specific resistance mechanisms, i.e., proteins that confer resistance for only one antibiotic resistance class; scenario 2 includes the first scenario and nonspecific resistance mechanisms, i.e., proteins that confer resistance to more than one antibiotic resistance class. In this second scenario (all proteins), the presence of two classes in the same genome does not necessarily mean they have been selected together. It may mean that a nonspecific mechanism that confers the resistance phenotype to more than one class. The resistance classes of disinfectants, glycopeptides, macrolides, and tetracyclines are present in more than 95% of the genomes for both scenarios. This suggests that the ubiquitous presence of these antibiotics, or their active residuals, leads to the selection of resistance and genome evolutions. The WHO considers glycopeptides and macrolides as critically important antibiotics because they are widely used in human medicine, sometimes as the only alternative [2]. Tetracyclines are also extensively used in veterinary and human medicine [37].

Concerning aminoglycosides and rifampicin, we have only identified resistance when considering the second scenario (all proteins; resistance in 887 genomes). This result suggests there might be a common nonspecific mode of resistance for antibiotics of these antibiotic classes, despite having different targets, since aminoglycosides act at the translation level and rifampicin at the transcription level.

We also characterized the amount of resistance classes present per genome, to analyze the levels of multi-resistance. Overall, we found almost ten antibiotic resistance gene classes in the genomes considering scenario 1 (specific proteins), and almost eleven considering scenario 2 (all proteins), which indicates a worrisome level of multi-drug resistance. The RefSeq reference genomes are primarily derived from sources of clinical interest, where the selective forces may favor multiple resistance.

Unfortunately, bacteria that are not genotypically resistant to antibiotics may, in some cases, survive in the presence of drugs, which further increases the problem of antibiotic resistance. One example is indirect resistance, in which bacteria sensitive to an antibiotic survive due to the detoxification of the medium by bacteria-producing enzymes that inactivate the antibiotic molecule [38,39]. Another example is bacterial persistence, in which bacteria survive the presence of antibiotics that interfere with cell division as they suspend their growth [40,41,42].

Recently, we have shown that antibiotic administration and the transfer of bacteria and their genes between individuals determine a positive correlation between resistance genes’ diversity and virulence genes’ diversity in human metagenomes [22]. In particular, the accumulation of resistance genes directly results from contact between individuals [22]. Therefore, when an individual whose microbiome has many resistance genes meets another individual, some microorganisms are shared along with the transfer of these genes to the second individual, increasing the diversity of resistance genes in the second individual’s microbiome. Our observation that several bacterial genomes contain many resistance genes suggests that a massive transfer of resistance genes can occur between people.

The use of antibiotics is still widespread, and the selective pressure for drug resistance is increasing. These drugs are frequently prescribed unjustifiably for reasons based on cultural values and psychological characteristics [43].

In addition, we have identified co-occurrences between many combinations of resistance classes. We detected more co-occurrences under the second scenario (all proteins) and on plasmids than in the first scenario (specific proteins). The association between two classes in the same genome was already expected to increase in the second scenario (all proteins), where we considered genes conferring resistance to more than one class. The higher number of co-occurrences in the plasmid may have resulted from the ability of several plasmids to transfer between bacteria. These mobile DNA elements are exposed to more selective pressures, and can also share insertion sequences that favor the accumulating of resistance to more antibiotic classes in a shorter period.

We analyzed in more detail the co-occurrences between classes of antibiotics of critical importance for the WHO, due to their importance to human health. As expected, there are more co-occurrences in the second scenario (all proteins) and slightly more in plasmids than chromosomes. All critically important resistance classes co-occur with a wide range of other classes, suggesting that there is a co-selection between resistance to critical and non-critical antibiotics. For example, the combinations quinolone—β-lactams resistances and colistin—β-lactams resistances co-occur above expected values on both chromosome and plasmid. These co-occurrences are worrisome because these antibiotic classes are one of the only resources for treating some bacterial infections. By taking one of the antibiotics that is not categorized as critical, we may also be co-selecting resistance to the critically important antibiotics if the two resistances are co-localized. For example, tetracyclines, considered by the WHO as highly important antibiotics and widely used for human medicine, co-occur in plasmids with all the critical classes (except for the β-lactams considering all the proteins).

Martiny et al. [31] have highlighted the co-occurrence of glycopeptides and macrolides in metagenomes sampled from mice, pigs, and humans. Our study found a higher-than-expected co-occurrence between these two resistance classes in both scenarios in plasmids but not in chromosomes. As mentioned above, the ability of plasmids to transfer between bacteria may explain this co-occurrence in plasmids.

With the same dataset used in the present study, Darmancier et al. [24] found an above-the-expected co-occurrence on chromosomes of type VII secretion systems and fusidic acid and pseudomonic acid. Under scenario 2 (all proteins), we observed a higher-than-expected co-occurrence on chromosomes of fusidic acid and the resistance classes trimethoprim, fosfomycin, and pseudomonic acid. This type of co-occurrence between several resistance classes, which in turn also have co-occurrences with virulence classes, demonstrates the existence of simultaneously multi-resistant and virulent profiles, linking antibiotic use to bacterial infection.

For the first time, an association of co-occurrences focusing on the priority antibiotic-resistant pathogen species identified by the WHO was studied. We found that the higher the priority, the higher the likelihood of co-occurrences on plasmids not observed on chromosomes. This result suggests that these resistances were more recently acquired. The presence of co-occurrences in elements that can be transferred between bacteria (sometimes between different bacterial species) intensifies the dissemination of resistance compared to vertical transmission (showing their epidemic potential). This can convert less critical species into antibiotic-resistant species, eventually making them critical. For instance, species categorized in priority 1 (critical) have resistance to carbapenems, which are part of the β-lactams. We identified co-occurrences for species in this category between the β-lactamase class and the 10 other classes. These co-occurrences suggest that these pathogens may tend to accumulate more antibiotic resistance genes in mobile genetic elements. This means that alternative antibiotics may be ineffective in treating bacterial infections caused by these species. Therefore, for infections with *Acinetobacter baumannii*, *Pseudomonas aeruginosa*, and *Enterobacteriaceae*, investing more in alternative treatments such as vaccines or phage therapy is essential. These types of treatment reduce the exposure of these pathogens to antibiotics, helping to reduce the spread of resistance and improve public health outcomes.

## 4. Materials and Methods

### 4.1. Dataset

For this work, we used a dataset previously prepared by our group [24]. Briefly, the protocol for preparing this dataset was:

1. Download the FASTA format files of the protein sequence of all complete bacterial genomes present in the National Center for Biotechnology Information (NCBI) Reference Sequence (RefSeq) database—16,622 genomes (from https://ftp.ncbi.nlm.nih.gov/genomes/refseq/bacteria/, accessed on 19 October 2020).
2. Format the protein sequence FASTA files belonging to the ResFinder database in the bash shell, using the BLAST manual as guidance. ResFinder is a comprehensive database divided into 17 classes of acquired genes and chromosomal mutations associated with antimicrobial resistance. The database was downloaded from https://bitbucket.org/genomicepidemiology/resfinder_db.git, and accessed on 30 October 2020 [44].
3. Running BLASTP using a bash script, and non-default parameters. qcov was set to 0.6 for coverage, the e-value was set to 1 × 10^−5^ and the output format was set to ‘6’, which gives a tabular format to the BLAST results. The BLAST+ executable package (version ncbi-blast-2.9.0+) was downloaded from the NCBI website (ftp://ftp.ncbi.nlm.nih.gov/blast/executables/blast+, accessed on 11 November 2019) [45].
4. A post-BLAST filter was applied to filter out hits with at least 30% identity. Each alignment that passed all filters was then divided into three categories based on its genomic location: (i) exclusively on the plasmid; (ii) exclusively on the chromosome; or (iii) both on the plasmid and on the chromosome.

Considering all the statistical processing that will be performed for this work, we did not include the genomic location “both on the plasmid and on the chromosome” due to the low number of counts obtained. Thus, at the end of this preparation, we have the antimicrobial classes per genome that had at least one hit, divided by genomic location. For each genome, we know which antimicrobial classes are present on the chromosome and the plasmid.

### 4.2. Co-Occurrence Analysis

To analyze whether each antibiotic resistance combination co-occurred more/less than expected, we constructed a matrix for the genomic location with as many rows and columns as the number of antimicrobial classes. Each cell of the matrix contains the number of genomes in which combination occurs.

We used RStudio to perform a binomial test (R function “binom.test”) to check if the value obtained for co-occurrence is different from the expected value (with a cutoff of α = 0.001). The binomial test has three inputs: *x*, which is the number of genomes in which resistance classes A and B co-occur; *n*, which is the total number of genomes with the genomic location we are using; and *p*, which is the expected probability that a genome has resistance A and B and is given by Equation (1).

(1) $$p=(\frac{Number\;of\;genomes\;with\;resistance\;A}{n})\times (\frac{Number\;of\;genomes\;with\;resistance\;B}{n})\;\;\;$$

Note that, when looking at the genomic location ‘chromosome’, *n* is the total number of genomes. However, when looking at the genomic location ‘plasmid’, *n* is the number of genomes containing plasmids with resistance genes.

The binomial test allows us to see if the co-occurrence is different from the expected value, but not if the value is above or below the expected value, nor how much the value deviates from the expected value.

To understand how much the correlation deviated from the expected value, we calculated the expected value by multiplying the expected probability (*p*) by the total number of genomes with that genomic location (*n*). To construct the heatmaps, we calculated the quotient of the observed value by the expected value.

## Figures and Tables

**Table 1:** Counts of the number of genomes in which each antibiotic resistance class is present.

Resistance Classes	Scenario 1 (Specific Proteins) Number of Genomes	Scenario 2 (All Proteins) Number of Genomes
Aminoglycoside	0	887
β-lactam	13,200	13,200
Colistin	5993	5993
Disinfectant	16,363	16,492
Fosfomycin	11,306	11,306
Fusidic acid	1065	1065
Glycopeptide	16,101	16,101
Macrolide	16,610	16,613
Nitroimidazole	2143	2143
Oxazolidinone	3691	16,445
Phenicol	12,682	15,657
Pseudomonic acid	7946	7946
Quinolone	5223	11,640
Rifampicin	0	887
Sulphonamide	14,940	14,940
Tetracycline	16,193	16,338
Trimethoprim	14,416	14,416

## Data Availability

Not applicable.
